# Some Changes That Take Place in and around the Pulp-Canal

**Published:** 1893-10

**Authors:** D. E. Caush

**Affiliations:** Brighton, England


### SOME CHANGES THAT TAKE PLACE IN AND AROUND THE PULP-CANAL.[1]

BY DR. D. E. CAUSH, BRIGHTON, ENGLAND.

During the microscopic examinations of exostosed human teeth, my attention was continuously drawn to certain alterations in the shape and structure of the pulp-canals; it was an unusual thing to find in an exostosed tooth a perfect pulp-canal, and this led me to the examination of a large number of these teeth. The subject has been under observation about five years, and the deductions drawn are from the examination of between two and three thousand teeth. I was led to the furtherance of this study on observing certain irregularities of outline in the pulp-canals of the teeth. In some cases I noticed here and there slight excavations at certain points of the canal, and from that stage was enabled to obtain microscopic slides illustrating the various stages of these changes, until the whole of the original outline of the pulp-canals had disappeared, leaving in its place a very irregular canal. It was also much enlarged, and it was observed that frequently in different directions the contour of the canal varied much, as the excavations may extend over a very small portion or they may continue in any given direction until a second canal is formed, frequently passing at right or acute angles to the original canal, continuing until it has passed through the dentine into the cemental tissue. These canals may be either simple or branched, or, instead of passing thus at angles to the pulp-canal, the two or three of an inferior or superior molar may be united together into one large irregular canal.

In all cases where this change has taken place the margins of the canals show a more or less irregular edge, produced by the semilunar excavations of the dentine.

The definite termination of the dentine, as seen in the pulp-canal of an ordinary tooth, has given place to the indefinite, and irregular margins are produced.

These semilunar excavations vary much in size, as well as in number, varying from a single slight dip or depression as found in the earlier stage to the numerous excavations producing the complex and irregular outline as seen in the advanced stage.

---

[1] Read before the World's Columbian Dental Congress, Chicago, August, 1893.

If, as is often the case, these teeth remain in position after these excavations have been produced, a second change more marked even than that of the excavation oftentimes follows, for in these depressions it is not at all unusual to have a deposition of new bony tissue, entirely different in microscopic structure from the dentine which surrounds it. In this new tissue we have no tubuli radiating to a given centre, as in the ordinary dentine, but instead we have a number of lacunæ and canaliculi, in character somewhat like those found in true cemental tissue. This tissue does not appear to be true bone, as it is very unusual to find any true Haversian system, even where there is comparatively a large amount of the new tissue deposited. The lacunæ as seen in this tissue are irregularly placed, as in the thickened cemental tissue, and vary much in number and position. They may be very closely packed with short canaliculi, or they may be scattered throughout the substance of the tissue, and have canaliculi of some length joining two or more of the lacunæ together.

As to how these changes are brought about is a subject of great interest, and we do well to carefully consider it.

The irregular margin and semilunar character of the edges give us a clue, and show at once that there must have been absorption going on in a greater or lesser degree, according to the amount of change that has taken place. This absorption may have commenced at different points of foci along the margin, and progressed till the whole of the margin is acted upon, or it may commence all along the edge of the canal at the same time.

It is evident (microscopically) that such an edge could not be produced by the addition of new tissue alone, as in cases where secondary dentine is deposited either as dentine of repair (in those cases where caries has almost penetrated to the pulp-canal, or as a result of repair following accidental penetration of the pulp-canal during the operation of preparing a cavity for filling), or as a result of pulp-calcification, for in either of the enumerated cases the microscopic structure of the tissue is quite different; in all these cases the new tissue is always added *directly* to the older tissue without any excavations or absorptions of the original margin, and the secondary dentine is deposited directly from the odontoblastic layer of the pulp. It would appear then that these excavations and this new tissue, cemental in character, could not be produced directly from the odontoblastic layer of the pulp in health, for we have seen under such circumstances, should there be any deposit, it would take the form of secondary dentine or pulp-calcification.

To understand the changes that have taken place in the teeth we must consider three things:

1. The change that takes place in the odontoblastic layer.
2. How the excavations along the margin of the original tissues are produced; and
3. The manner in which the new tissue is deposited.

It may help us to understand these changes if we devote a short time to the careful examination of the exterior surface of an exostosed tooth. We will take for our examination a tooth where there has been acute inflammation of the membrane, and then a time of rest. On examining such teeth, the first thing to which our attention will be drawn is the thickened condition of the membrane, which may appear two or three times as thick as in health, and at certain places under the membrane we may frequently find excavations going on and giant-cells in position; again, in another place in this root we may discover some older excavations, and a layer of new tissue filling up the excavations and increasing the size of the exostosed tooth by the additional tissue formed; in fact, the three stages of the development of cemental tissue in exostosis.

Keeping these changes before us we will endeavor to follow the history of the changes that take place in the pulp-canal.

The first we have to consider is that which occurs in the layer of odontoblasts or lining cells of the pulp-canal. These cells are the points of attachment, too, and the source of nourishment from the pulp to the dentine, the connecting link between the hard calcified dentine and the soft vascular tissue and nerve-matter that forms the pulp; it is from this layer that the secondary dentine is formed, but under certain circumstances the character of the cells forming this layer changes, both in shape and size. Thus, if by such cause as congestion or slight inflammation of the pulp, a greater amount of blood is carried to the blood-vessels in the pulp, these cells appear to lose their original shape, and (instead of being elongated with processes penetrating into or passing between the tubuli) swell up, the nuclei becoming active, and new cells are rapidly formed by cell-division. As these new cells continue to form, a layer like the alveolar dental membrane in appearance is produced, and this newly-formed layer presses upon the dentine. The cells in juxtaposition with the dentine begin to absorb the latter, and thus obtain more room for further development. This goes on as long as there is an abnormal blood-supply to the pulp, and thus, owing to either a long or short time of pressure upon the dentine, caused by the excess of formative material brought to the cells by the increased blood-supply, many or few excavations may be produced, as in the case of chronic inflammation of the pulp we get many excavations, and oftentimes these penetrate more deeply into the original tissue. Or should there be any imperfectly-calcified tissue surrounding the pulp-canal, at that point the giant-cells will penetrate more easily and the excavations will increase in that direction, and in some cases produce canals that pass out of the pulp-canal at various angles. These excavations may grow continuously, or there may be times of quiescence as well as times of activity, while these terms of activity or rest may correspond with similar changes on the external surface of the tooth and produce layers of cemental tissue. Should the tooth be extracted about this period, nothing but the excavations or the thickened membrane will appear in the pulp-canal.

These changes, as we have seen, are produced by those that have taken place in the odontoblastic layer of the pulp-canal. If the tooth is not extracted the whole of the pulp becomes more or less congested and altered in character; the ondontoblastic layer being destroyed, there is little or no intercommunication with the dentine by the tubuli, and at this stage one of two things may occur:

1. The blood-supply becomes normal, the congestion temporarily passes away, and for a short time the tooth is tender to the touch as if there had been periostitis, and from this stage it gradually becomes comfortable, and remains so until the inflammation again commences, or,—

2. The giant-cells change their character and become formative cells, producing as a result of calcification a fresh tissue in the canal containing a number of lacunæ with their canaliculi. In this tissue a new tissue is formed in the enlarged pulp, in character and in microscopic structure resembling cementum, developed also from a membrane similar in character to the alveolar-dental membrane; and on considering the close relationship during development of the two tissues (dentine and cementum), it does not appear to be at all difficult to follow the various stages that take place in the production of this new tissue. These changes are not confined to the human subject. I have found the various stages in the incisors of the horse, though the molars are all free from any of the various changes. I have also found the incisors of the horse exostosed, as well as the enlargement of the pulp-canal and the new tissue deposited in the excavations.

